# Understanding the evolving treatment landscape of hidradenitis suppurativa: An analysis of All of Us

**DOI:** 10.1371/journal.pone.0331032

**Published:** 2025-08-22

**Authors:** Aditya K. Gupta, Vasiliki Economopoulos, Paradi Mirmirani, Renata Magalhaes

**Affiliations:** 1 Mediprobe Research Inc., London, Ontario, Canada; 2 Division of Dermatology, Department of Medicine, Temerty Faculty of Medicine, University of Toronto, Toronto, Ontario, Canada; 3 Department of Dermatology, The Permanente Medical Group, Vallejo, California, United States of America; 4 Department of Dermatology, University of California, San Francisco, San Francisco, California, United States of America; 5 Department of Dermatology, Case Western Reserve University, Cleveland, Ohio, United States of America; 6 University of Campinas, Campinas, São Paulo, Brazil; Istituto Dermopatico dell’Immacolata (IDI)-IRCCS, ITALY

## Abstract

**Background:**

Hidradenitis suppurativa (HS) is a chronic skin condition with significant burden for affected patients. The development of new therapies in the last decade has brought hope for better patient outcomes, but understanding the use of these new drug classes is necessary to ensure patient access to care.

**Methods:**

We analysed data from 2636 HS patients within NIH’s All of Us research program from June 2017 and October 1, 2023 to examine the likelihood of patients receiving certain classes of drugs based on socio-demographic factors and comorbid health conditions, as well as how these drug classes impact quality of life. We also examined trends in the number of prescriptions over time.

**Results:**

Antibiotics were most frequently prescribed, with higher numbers administered to females. Small molecule inhibitors and biologic medications were prescribed at low levels. We found that socio-demographic factors such as ethnicity, income and insurance provider influence the types of drugs patients are most likely to receive, with African American individuals more likely to receive antibiotics and immunosuppressive drugs and less likely to receive small molecule inhibitors. We also found that comorbid conditions significantly influence the likelihood of patients receiving specific drugs, with higher odds of receiving biologics if a patient has a comorbid rheumatic/autoimmune disorder. Antibiotics and immunosuppressive drugs were associated with less favourable quality of life measures, as well as a higher likelihood of anxiety and depression.

**Conclusions:**

This work uncovers the varied landscape of HS treatment in the US and highlights the factors that influence treatment choices and how these treatments impact quality of life. It also provides an understanding of the social disparities that some populations face when accessing HS care, informing future decisions and practices to reduce these inequities.

## Introduction

Hidradenitis suppurativa (HS) – which presents as painful inflammatory nodules, abscesses and draining fistulas – has significant physical and psychological burden on patients, frequently leading to a decreased quality of life [1]. Diagnosis of HS is frequently delayed and tends to occur approximately 7–10 years after onset. This delay can lead to improper or a lack of care, further worsening the patient’s condition [2]. HS also tends to associate with several comorbid conditions, including but not limited to: Crohn’s disease, polycystic ovary syndrome, rheumatoid arthritis and spondyloarthritis [3–6]. Treatment for this condition generally relies on antibiotics, anti-inflammatory/immunosuppressive medications and surgical procedures [2,7,8]. However, over the past decade there have been significant advances in the treatment of HS with the advent of biologic medications and small molecule inhibitors. Currently, adalimumab, secukinumab and bimekizumab are the only FDA approved biologics for the treatment of HS [1,2], with additional biologics and small molecule inhibitors currently in clinical trials [2,8,9].

With the development of these newer therapeutic options for patients, it is important to assess how HS patients in the general population are able to access this type of care. Of note, HS occurs more frequently in patients that are female and of Black/African American descent [2,3,5,10,11]. Historically, individuals from marginalized social/demographic groups have had limited access to dermatologic care when compared to the general population [12]. We wanted to address this issue by studying the management of HS within the context of the National Institute of Health’s (NIH) All of Us (AoU) research program [13–15]. This program aims to create a diverse patient dataset to further support precision medicine and research, particularly in under-represented communities. Studies have used the AoU to examine the effect of social/demographic factors on the diagnosis and treatment of dermatological disorders [10,16,17].

The main objective of this study was to evaluate therapeutic trends within HS, particularly the uptake of newer therapeutic agents, such as biologics and small molecule inhibitors and how these medications may be influencing patients’ quality of life. We also aimed to determine how various demographic factors, as well as the presence of comorbid conditions, impact the choice of treatment in these patients.

## Methods

The NIH’s AoU controlled tier dataset version 8 was used for all analyses performed. The All of Us Research Program data is distributed on a cloud-based data platform, enabling qualified researchers to run analysis without downloading the data. The Registered and Controlled Tiers allow for both Web-based access and computational analysis for research studies but do not include explicit identifiers (e.g., names, personal identifying numbers such as SSN sand MRNs). Ethics approval requirements are not applicable to this research. As an anonymized database with no potential to re-identify subjects, access to research data though the All of Us ‘Controlled Tier’ database does not qualify as research involving human subjects that would require prior ethics approval for use, per the All of Us Institutional Review Board, the US Department of Health and Human Services Code of Federal Regulations 45 CFR 46 definitions and Canadian Tri-Council Policy Statement 2 (2022) guidelines. For more details on the All of Us Research Program, please visit https://www.researchallofus.org/. Additionally, researchers at partnered institutions must complete data usage and privacy training before access can be granted to the dataset.

In our cross-sectional study, we accessed survey and electronic health record (EHR) data from 2636 participants that had been diagnosed with HS and had enrolled in the program between June 2017 and October 1, 2023. We collected data on the types of medications prescribed by demographic factors (age, sex, ethnicity, income, education), insurance status and type, comorbid conditions and quality of life measures, as listed in Table 1. Only oral and injection medications were evaluated. All concept identification codes (concept IDs) used to identify medications, conditions and survey responses are found within the Observational Medical Outcomes Partnership (OMOP) framework and listed in S1 Table. A summary of our aggregate data can be found in S2 Table, with the full AoU dataset available on the program’s website after researcher registration (https://www.researchallofus.org).

**Table 1:** List of medications, demographic factors and comorbid conditions.

Medication (by drug class)	Demographic factors	Comorbid conditions	Quality of life: questions and answers
Biologics			Level of pain
* Guselkumab*	Age	Crohn’s disease	Scale from 0 to 10
* Risankizumab*	Income	Hypertension (any type)	Level of fatigue
* Certolizumab pegol*	Education	Poly-cystic ovary syndrome (PCOS)	None, Mild, Moderate, Severe or Very Severe
* Adalimumab*	Insurance type	Psoriasis	Ability to carry out social responsibilities
* Secukinumab*	*Medicaid*	Spondyloarthritis (SpA)	Completely, Mostly, Moderately, A Little, Not At All
* Infliximab*	*Medicare*	Rheumatoid arthritis	Satisfaction with social relationships
* Usteskinumab*	*Employer or union supplied*	T2 diabetes mellitus	Excellent, Very Good, Good, Fair, Poor
* Brodalumab*	*Independently Purchased*	Ulcerative colitis	Ability to carry out everyday activities
			Completely, Mostly, Moderately, A Little, Not At All
Small molecule inhibitors	Race/ethnicity		Overall quality of life
* Anakinra*	*White*		Excellent, Very Good, Good, Fair, Poor
* Apremilast*	*Black/African American*		Anxiety
* Upadacitinib*	*Hispanic*		Depression
* Roflumilast*			
* Etanercept*			
Antibiotics			
* Clindamycin*			
* Erythromycin*			
* Moxifloxacin*			
* Tetracycline*			
* Ertapenem*			
* Ceftriaxone*			
* Doxycyline*			
Traditional immunosuppressive medications			
* Triamcinolone*			
* Metronidazole*			
* Dapsone*			

All analyses were performed within the AoU’s Researcher Workbench with the SAS Studio application (Statistical Analysis Software, SAS Institute, Cary, North Carolina). We used logistic regression models with Wald’s Chi-square test to determine the odds that a particular patient population received a specific class of medications. The significance level for all comparisons was set to a = 0.05. All odds ratios (OR) were adjusted for sex and age where applicable.

The privacy of individual participants in the AoU program must be upheld, requiring us to not display any patient numbers that are less than 20, and to not display other data in such a way that data with less than 20 observation can be identified.

## Results

To ensure consistency of our analysis with previously published work, we analysed the odds of diagnosis by sex at birth and ethnicity. Similar to previous studies, we found that Black/African American individuals (OR (95% CI): 2.41 (2.20–2.64); p < 0.0001) and females (OR (95% CI): 2.53 (2.28–2.81); p < 0.0001) were more likely to be diagnosed with HS [3,5,10,11,18].

### Prescriptions trends in HS patients

**Fig 1:**
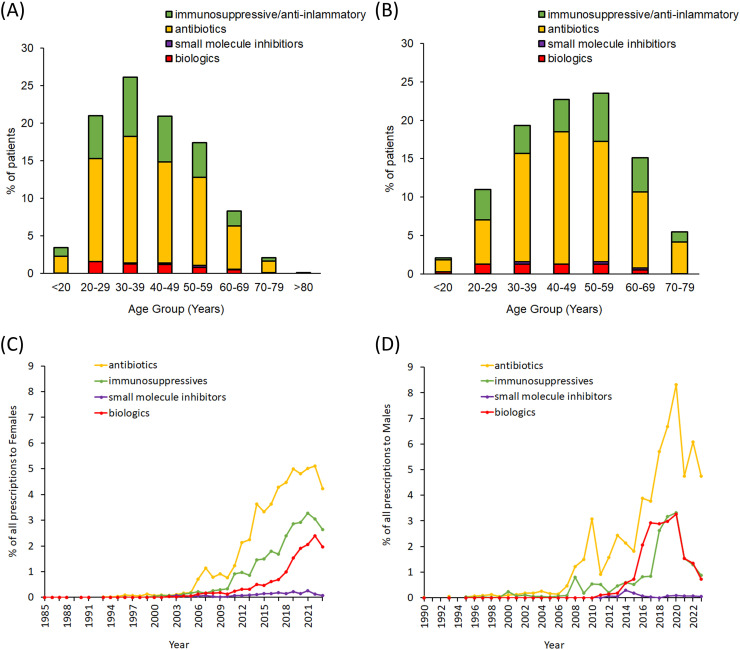
Prescription trends in HS patients. Stacked bars represent the proportion of patients in each age group receiving each drug class in **(A)** Females and **(B)** Males. Prescription rates over time for each drug class are shown for (C) females and (D) males.

We determined the proportion of patients receiving antibiotics, traditional immunosuppressive drugs, biologics and small molecule inhibitors by both age and gender (Fig 1A and 1B). The majority of patients in each age group for both males and females received antibiotics, while very few have received small molecule inhibitors. When we examined the rates of prescriptions over time (Fig 1C and 1D) we found that the levels of prescriptions for all drug classes has tended to increase over time. In both males and females, antibiotics are prescribed most frequently, while small molecule inhibitors have been prescribed the lowest levels.

**Fig 2:**
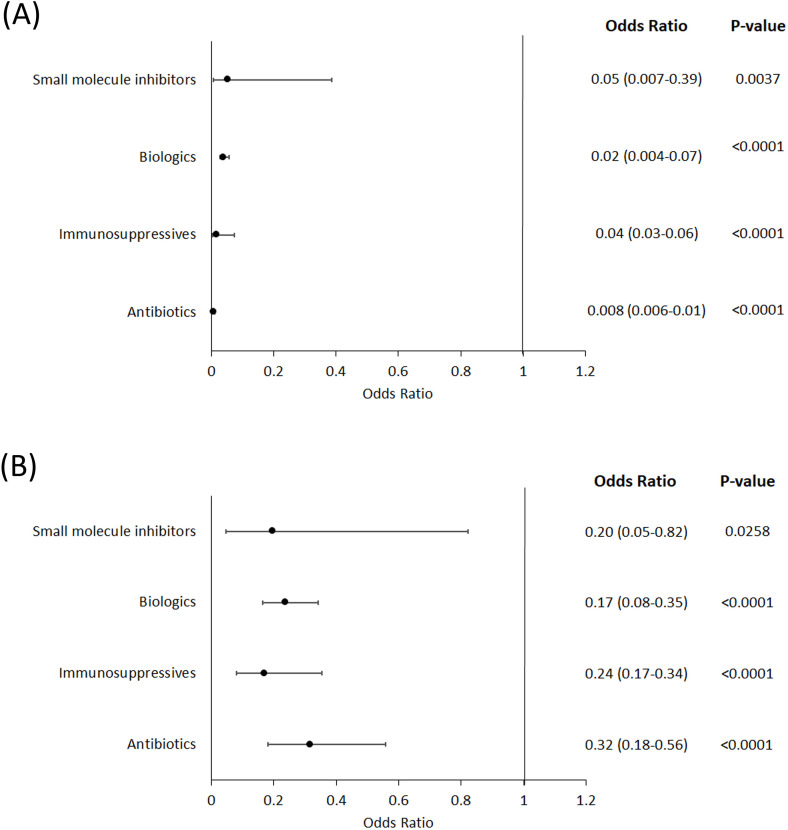
Likelihood of HS patients receiving a particular class of drug by age group. **(A)** HS patients under the age of 18 years compared to those aged 18 to 64 years. **(B)** HS patients over the age of 65 years compared to those aged 18 to 64 years. The reference group for odds ratios that are presented in both panels is HS patients aged 18 to 64 years of age.

In addition to the age breakdown shown in Fig 1, we stratified patients into under the age of 18 (pediatric), between 18 and 64 years of age (mid-age) and over the age of 65 (elderly) to further quantify differences in prescription rates (Fig 2). In the pediatric population (Fig 2A) we observed that these patients are less likely to receive systemic antibiotics (OR (95% CI): 0.008 (0.006–0.01), p < 0.0001), immunosuppressives (OR (95% CI)0.04 (0.03–0.06), p < 0.0001), biologics (OR (95% CI): 0.02 (0.004–0.07), p < 0.0001) and small molecule inhibitors (OR (95% CI): 0.05 (0.007–0.39), p = 0.0037) when compared to mid-age HS patients. In elderly patients (Fig 2B), we found that antibiotics (OR (95% CI): 0.32 (0.18–0.56), p < 0.0001), traditional immunosuppressives (OR (95% CI): 0.24 (0.17–0.34), p < 0.0001), biologics (OR (95% CI): 0.17 (0.08–0.35), p < 0.0001) and small molecule inhibitors (OR (95% CI): 0.20 (0.05–0.82), p = 0.0258) were less likely to be prescribed when compared to mid-age HS patients.

**Fig 3:**
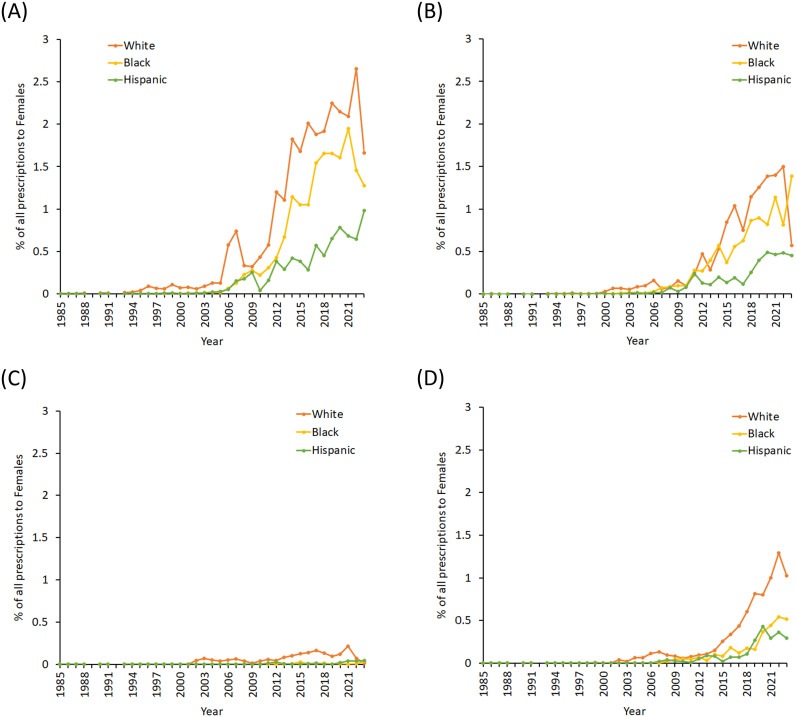
Prescription rates over time in females by race/ethnicity for (A) Antibiotics, (B) Immunosuppressives, (C) Small molecule inhibitors and (D) Biologics.

When we further break down the trends observed in Fig 1C and 1D, we found variations in the rates of prescriptions attributable to each ethnicity, particularly in females (Fig 3). White females appeared to have the highest rates of prescriptions in each of the drug classes that we examined, even after accounting for differences in the proportion of patients from each ethnicity. In males, there does not appear to be any meaningful differences in the proportion of prescriptions by ethnicity (S1 Fig).

### Impact of demographic factors on drug choice

**Fig 4:**
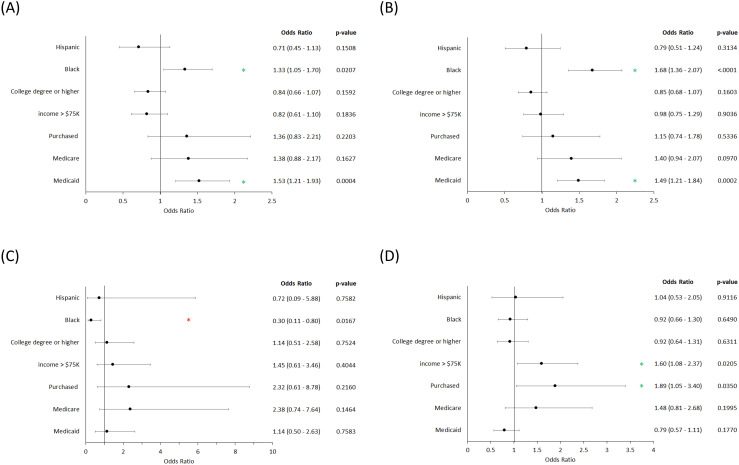
Effect of demographic factors on the odds of patients being prescribed (A) antibiotics, (B) immunosuppressives, (C) small molecule inhibitors and (D) biologics. All odds ratios are derived from multivariate models and are age and sex adjusted. Green * - significant positive association. Red * - significant negative association.

We examined the influence of various demographic factors on the likelihood of patients receiving certain classes of treatments (Fig 4). All odds ratios shown within Fig 3 are derived from a multivariate model and both age and sex adjusted.

When we examined patient ethnicity, we found that there was a higher likelihood of patient of Black/African American descent receiving antibiotics (Fig 4A, OR (95% CI): 1.33 (1.05–1.70)) and traditional immunosuppressive medications (Fig 4B, OR (95% CI): 1.68 (1.36–2.07)) when compared to White patients. At the same time, we found that these patients also less likely to receive small molecule inhibitors (Fig 4C, OR (95% CI): 0.30 (0.11–0.80)). We found that there is no difference in the likelihood of receiving biologics between the different ethnicities (Fig 4D).

We next examined the impact of income and education on the treatment choice, we found that biologics are more likely to be prescribed to those that earn over $75,000/year (Fig 4D OR (95% CI): 1.60 (1.08–2.37). We did not observe an effect for education in any of the drug classes. We evaluated the impact of different health insurance plans/providers. For both antibiotics and immunosuppressive drugs, patients whose coverage through Medicaid were more likely to receive these classes of drugs (Fig 4A, OR (95% CI): 1.53 (1.21–1.93)); Fig 4B, OR (95% CI): 1.49 (1.21–1.84)) than those with employer or union provided coverage. Patients who had purchased their own health plans were more likely to receive biologics compared to employer or union provided plans (Fig 4D, OR (95% CI): 1.89 (1.05–3.40)).

### Impact of comorbid conditions on drug choice

**Fig 5:**
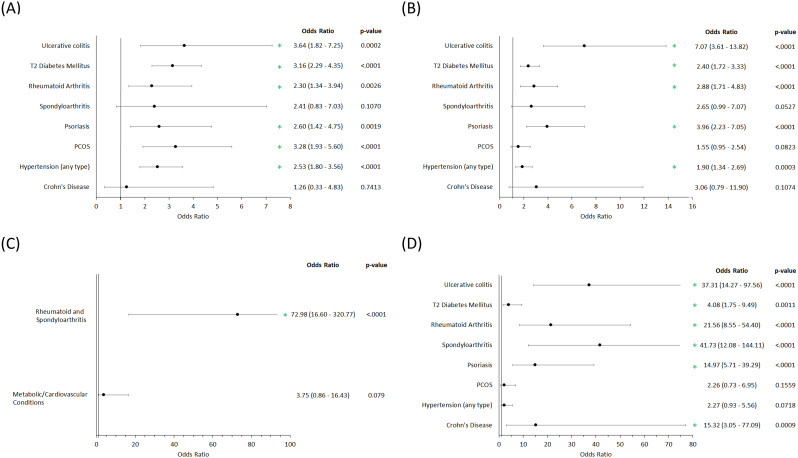
Effect of comorbid conditions on choice of treatment for (A) antibiotics, (B) immunosuppressives, (C) small molecule inhibitors and (D) biologics. All odds ratios are derived from multivariate models and are age and sex adjusted. Green * - significant positive association.

We investigated how the presence of various comorbid conditions impacts the use of each drug class (Fig 5). The odds ratio and p-value for each comparison is shown in a table next to the graph in each panel. We found that antibiotics (Fig 5A) were more likely to be prescribed in patients with comorbid hypertension, PCOS, psoriasis, rheumatoid arthritis, T2 diabetes mellitus and ulcerative colitis when compared to patients with no additional comorbidity. A similar pattern was observed when we examined the use of traditional immunosuppressive drugs (Fig 5B), where comorbid hypertension, psoriasis, rheumatoid arthritis, T2 diabetes mellitus and ulcerative colitis were associated with increased immunosuppressive drug use. Drugs within the biologics drug class (Fig 5D) were also found to be prescribed more frequently in patients with concurrent Crohn’s disease, psoriasis, spondyloarthritis, rheumatoid arthritis, T2 diabetes mellitus and ulcerative colitis. Patients with comorbid rheumatoid arthritis and spondyloarthritis were significantly more likely to receive small molecule inhibitors (Fig 5C).

### Impact of drug class on quality of life and mental health

We investigated the impact of antibiotics, immunosuppressives, biologics and small molecule inhibitors on the reported quality of life and mental health (Table 2). We found that HS patients prescribed antibiotics had higher levels of pain (OR (95% CI): 1.26 (1.01–1.52), p = 0.0174), less satisfaction in social relationships (OR (95% CI): 1.48 (1.22–1.81), p < 0.0001) and reduced ability to carry out everyday activities (OR (95% CI): 1.34 (1.05–1.70), p = 0.0184). We also found that immunosuppressive drugs were associated with higher pain levels (OR (95% CI): 1.30 (1.09–1.53), p = 0.0027), reduced ability to carry out social responsibilities (OR (95% CI): 1.23 (1.01–1.49), p = 0.0382) and everyday activities (OR (95% CI): 1.32 (1.07–1.61), p = 0.0088), lower satisfaction with social relationships (OR (95% CI): 1.37 (1.15–1.62), p = 0.0003) and reduced overall patient reported quality of life (OR (95% CI): 1.35 (1.13–1.62), p = 0.0010). We did not observe a significant impact of biologics or small molecule inhibitors on the quality of life of HS patients.

**Table 2:** Impact of drug classes on various quality of life measures and mental health.

	Level of Pain	Level of Fatigue	Carry out social responsibilities	Satisfaction with social relationships	Carry out everyday activities	Overall quality of life	Anxiety	Depression
	5 or greater (worse pain)	Moderate, severe and very severe	Fair and poor	Fair and poor	Fair and poor	Fair and poor	Diagnosis	Diagnosis
**Antibiotics**								
OR* (95% CI**)	1.26 (1.01-1.52)	1.06 (0.88-1.26)	1.23 (0.99-1.54)	1.48 (1.22-1.81)	1.34 (1.05-1.70)	1.19 (0.97-1.46)	1.61 (1.34-1.94)	1.65 (1.37-1.98)
p-value	**0.0174**	0.5547	0.0625	**< 0.0001**	**0.0184**	0.091	**< 0.0001**	**< 0.0001**
	Associated with higher pain			Associated with worse social activity	Associated with worse daily functioning		Associated with anxiety	Associated with depression
**Biologics**								
OR (95% CI)	0.923 (0.68-1.25)	1.01 (0.75-1.36)	1.23 (0.88-1.70)	1.30 (0.96-1.75)	0.98 (0.68-1.41)	1.03 (0.75-1.41)	1.01 (0.74-1.37)	0.85 (0.63-1.16)
p-value	0.6091	0.935	0.224	0.0879	0.9091	0.8644	0.9721	0.3074
**Immunosuppressives**								
OR (95% CI)	1.30 (1.09-1.53)	1.08 (0.92-1.27)	1.23 (1.01-1.49)	1.37 (1.15-1.62)	1.32 (1.07-1.61)	1.35 (1.13-1.62)	1.43 (1.21-1.70)	1.68 (1.41-2.00)
p-value	**0.0027**	0.3544	**0.0382**	**0.0003**	**0.0088**	**0.0010**	**< 0.0001**	**< 0.0001**
	Associated with higher pain		Associated with worse social functioning	Associated with worse social activity	Associated with worse daily functioning	Associated with worse quality of life	Associated with anxiety	Associated with depression
**Small Molecule Inhibitors**								
OR (95% CI)	1.01 (0.54-1.86)	1.48 (0.79-2.77)	0.67 (0.35-1.30)	0.64 (0.35-1.19)	0.86 (0.44-1.68)	0.86 (0.47-1.57)	1.09 (0.59-2.04)	0.68 (0.73-1.23)
p-value	0.9879	0.2257	0.2394	0.1588	0.6556	0.6223	0.7771	0.2011

*Odds Ratio.

**95% Confidence Interval.

We also examined the impact of these drugs on the likelihood of anxiety and depression in HS patients (Table 2). We again found that antibiotics and immunosuppressives are associated with increased likelihood of anxiety (OR (95% CI): 1.61(1.34–1.94), p < 0.0001 for antibiotics; 1.43(1.21–1.70), p < 0.0001 for immunosuppressives) and depression (OR (95% CI): 1.65(1.37–1.98), p < 0.0001; 1.68(1.41–2.00), p < 0.0001; respectively). We did not observe any significant association with biologics and small molecule inhibitor for both anxiety and depression.

## Discussion

The work we have presented provides valuable insight into the treatment landscape of HS. By using data from the AoU, we have been able to identify treatment trends and how patients’ health status, as well as social disparities may impact patient care. We build onto the work of Desir, et al., Pathak, et al., and Roster, et al. as well as others who have examined the influence of demographic factors on HS diagnosis and the association of HS with other comorbid conditions [3,11,18,19].

In our current work, we found that the rates of prescriptions of the different drug classes (antibiotics, immunosuppressives, biologics, small molecules) vary by age group in both males and females (Fig 1A and 1B); we also observed that the peak age range for receiving prescription treatment was lower in females (30–39 years of age) than in males (40–49 and 50–59 years of age). When we examined the proportions of prescriptions in each age group, we found that prescriptions for antibiotics accounted not only for the majority of the prescriptions, but also appears to be responsible for the overall skewed trend where they were prescribed more frequently in females than males, and in the younger age group in females. Compared to antibiotics, traditional immunosuppressive drugs were prescribed at modest levels while biologics and small molecule inhibitors were prescribed at low levels across age groups in both males and females.

We stratified patients into pediatric (under the age of 18), mid-age (18–64 years of age) and elderly (over 65 years of age) to further quantify differences in prescribing rates that may arise from the specific needs of these patient groups. Using logistic regression models, we found that patients in the pediatric (Fig 2A) and elderly populations (Fig 2B), when compared to the mid-age group, had significantly reduced likelihood of receiving antibiotics, immunosuppressives, biologics and small molecule inhibitors, which is reflected within Fig 1A and 1B. The level of prescriptions of both biologics and small molecule inhibitors is at a much lower level than the other drug classes, which may be due to their relatively recent development. Additionally, the lower rates of prescriptions to elderly HS patients may be due to these patients potentially having more a complex health status compared to the mid-age HS patients. Also, pediatric HS patients may not be receiving biologics and small molecule inhibitors as frequently due to these classes not necessarily having approval in this age group or practitioner hesitancy due to lack of efficacy and safety data in this population [20,21].

When we extracted the total prescription rates over time for each class of drugs (Fig 1C and 1D), we observed a similar pattern where antibiotics have the highest usage in both males and females, while the use of small molecule inhibitors within HS patients remained low over time. Overall, the usage of antibiotics has increased over time. The lower rates of biologic and small molecule usage could be attributed to the more recent development of these drug classes. Biologics such as adalimumab which was only approved for use in HS in 2015, while secukinumab was approved more recently in 2023 and bimekizumab in 2024 [22]. In this study, we have examined eight different biologics (Table 1) indicating that some of these drugs are possibly being used off-label to treat HS.

We next assessed how different demographic factors, including gender, ethnicity, education, income and health insurance impact how patients are able to access treatment (Figs 3 and 4). Using a multivariate model to remove potential confounding effects we found that there are differences in drug prescription patterns. Specifically, in our temporal analysis, the highest levels of prescriptions were given to White females across all drug classes (Fig 3). In our multivariate model (Fig 4), Black individuals were more likely to receive antibiotics and traditional immunosuppressive drugs compared to Whites, while being less likely to receive small molecule inhibitors. We also found in our multivariate model that patients with Medicaid coverage were 1.53 and 1.49 times more likely on average to receive antibiotics and immunosuppressive drugs, respectively (Fig 4A and 4B), while those that had purchased their own insurance plans or made over $75,000/year were 1.89 and 1.60 times on average more likely to receive biologics, respectively (Fig 4D). These results highlight not only the existence of disparities, but that the nature of these disparities is complex and impacts patient outcomes.

We examined how comorbid health conditions influence the use of our drug classes of interest (Fig 5). In this analysis, we once again used a multivariate model to account for the potential influence of multiple comorbid conditions within a patient. We found that for antibiotics, the presence of hypertension, T2 diabetes mellitus, rheumatoid arthritis, psoriasis, PCOS and ulcerative colitis was associated with increased likelihood of patients receiving these medications. We observed a similar pattern for traditional immunosuppressive drugs.

Interestingly, rheumatoid arthritis and spondyloarthritis were associated with an increased likelihood of receiving small molecule inhibitors. We observed strong associations between biologics and known autoimmune/inflammatory disorders, specifically Crohn’s disease, ulcerative colitis, rheumatoid arthritis, spondyloarthritis, psoriasis and T2 DM. Currently, many of these disorders can be treated with biologics, leading us to speculate that much of the usage in HS patients is to address the comorbid condition primarily, with possible HS improvement/ management as a secondary consequence. However, the presence of one of these other conditions may make it easier for clinicians to obtain insurance approval for the prescription. Additionally, it is important to note that not all biologic usage is within patients with comorbid conditions, indicating that some biologics are in fact being used off-label to treat these patients [22].

An important aspect of HS is its impact on the quality of life and mental health of those affected. We investigated how quality of life may vary for those prescribed antibiotics, immunosuppressives, biologics and small molecule inhibitors. As these newer biologics and small molecule inhibitors have higher costs and accessibility to these drugs may be dependent on a patient’s income and insurance, it is important to know if and how these drug classes impact patients’ overall wellbeing. In Table 2, we present the results of our multivariate regression model where we have examined the impact of each drug class relative to no systemic medication on the likelihood of less favourable quality of life measures as well as the association of anxiety and depression with each drug class. We found that antibiotics were associated with worse patient reported pain levels, social interaction and a greater impairment in carrying out everyday activities. We also found that patients that were prescribed antibiotics were more likely to have anxiety or depression. A similar trend was observed for immunosuppressive drugs, where patients were more likely to experience higher levels of pain, a reduced ability to carry out social and everyday responsibilities, poor social interactions and relationships and an overall reduced patient reported quality of life. Similar to antibiotics, patients receiving immunosuppressives were more likely to experience anxiety or depression.

However, this trend is not observed with those patients on biologics and small molecule inhibitors. These patients have a similar likelihood of less favourable quality of life and mental health outcomes as those HS patients who did not receive any systemic medications. Importantly, the HS patients that have not received systemic therapies may have received topical therapies which are typically used in more mild cases where an improved quality of life would be expected compared to those with more significant disease [23]. We see this reduced quality of life in those patients with systemic antibiotics and immunosuppressives where it would be reasonable to except more significant disease. As biologics and small molecule inhibitors are typically indicated for more significant disease [24], our observation of similar likelihoods for the quality of life and mental health outcomes indicates that these medications are associated with improved overall health for these patients.

Our work may help clarify the role of these different drug classes within the treatment of HS and how access to these drugs may differ based on demographic factors and could influence patient outcomes. Traditionally, treatment for HS involves the use of antibiotics and immunosuppressive drugs however, the improved efficacy and safety profile observed with biologics and small molecule inhibitors has the potential to greatly improve patient outcomes, particularly in those patients with more severe disease.

There are several limitations to the work that we have presented which need to be considered. Firstly, AoU data is curated through electronic health records, which may not be fully accurate and complete. Cai, et al. have demonstrated the existence of inaccuracies within the AoU dataset by examining the rate of sex incongruent conditions being listed within EHRs [25]. Even though the rates of these incongruencies were low – less than 1%, there is the potential for these EHR errors to impact results, particularly when small groups of patients are studied [25]. Additionally, work by Bell, et al. has demonstrated that when patients examine their own EHR data, they identified errors in 21.1% of cases [26]. Klinger, et al. found inaccuracies when they examined the accuracy of ethnicity and language preference within EHR data compared to self-reported information [27]. These inaccuracies may arise when patients do not share all information with their healthcare provider, but also from the healthcare provider not correctly coding the patient’s information within the EHR system [28]. These inaccuracies, as discussed above, have the potential to skew the results of retrospective studies of EHR data. Second, the AoU program suppresses some information within the dataset in order to protect the privacy of participants. Specifically, we are not able to access information on the specialty of healthcare practitioner that is providing treatment to patients, which is an important factor in determining access to care.

Thirdly, we have only included medications administered orally and by injection. We chose to focus on these medications as they are more likely to be administered in patients with more severe and active disease. By excluding topical medications, we do not have information about the potential treatment of mild disease, where patients may also face differences in access to and choice of treatment. Even with the limitations listed above, the real-world data that can be extracted from EHRs enables practical insight into the ever-changing landscape of patient health management.

## Conclusions

This work has provided valuable insight into the current state of HS treatment, encompassing both traditional treatment approaches, as well as newly emergent treatment options. We have demonstrated that there are distinct differences in the treatment of HS dependent on a patient’s social and overall health status. We have also demonstrated a measurable effect of newer biologics and small molecule inhibitors on patients’ quality of life. Our work highlights the complex interactions between social factors, prescribed treatment and quality of life. We have also illustrated how treatment decisions in HS may be influenced by the patient’s overall health status, where certain conditions, particularly rheumatic and auto-immune conditions, may dictate or facilitate the treatment a patient receives for the management of HS.

## Supporting information

S1 Table All concept IDs used within the AoU program. (DOCX)

S2 Table Summary data for HS population within the AoU program. (DOCX)

S1 Fig Prescription rates over time in males by race/ethnicity. (A) Antibiotics, (B) Immunosuppressives, (C) Small molecule inhibitors and (D) Biologics.
(TIF)

S1 File STROBE/RECORD Checklist. (DOCX)
