# Supplementary material for: Understanding the evolving treatment landscape of hidradenitis suppurativa: An analysis of All of Us
Source: PLoS One. 2025 Aug 22;20(8):e0331032. doi: 10.1371/journal.pone.0331032 (PMC12373187; doi:10.1371/journal.pone.0331032)
Supplement: S1 Table — (DOCX) [file pone.0331032.s001.docx]

**S1 Table** – Concept IDs for all items used within the All of Us research program

| **Item** | **Concept ID** |
| --- | --- |
| Income: What Annual Income | 1585375 |
| Annual Income: less 10k | 1585376 |
| Prefer Not To Answer | 903079 |
| Annual Income: 10k 25k | 1585377 |
| Annual Income: 50k 75k | 1585380 |
| Annual Income: 35k 50k | 1585379 |
| Annual Income: 25k 35k | 1585378 |
| Annual Income: 100k 150k | 1585382 |
| Annual Income: 75k 100k | 1585381 |
| Annual Income: more 200k | 1585384 |
| Annual Income: 150k 200k | 1585383 |
| PMI: Skip | 903096 |
|  |  |
| Hispanic or Latino | 38003563 |
| No matching concept | 0 |
| Not Hispanic or Latino | 38003564 |
| PMI: Prefer Not To Answer | 903079 |
| PMI: Skip | 903096 |
| White | 8527 |
| Black or African American | 8516 |
| Race/Ethnicity: None Of These | 1586148 |
|  |  |
| Education Level: What Highest Grade | 1585940 |
| Highest Grade: Advanced Degree | 1585948 |
| Highest Grade: College Graduate | 1585947 |
| Highest Grade: Twelve Or GED | 1585945 |
| Highest Grade: College One to Three | 1585946 |
| Highest Grade: Nine Through Eleven | 1585944 |
| Highest Grade: Five Through Eight | 1585943 |
| Highest Grade: One Through Four | 1585942 |
| Prefer Not To Answer | 903079 |
| Highest Grade: Never Attended | 1585941 |
| PMI: Skip | 903096 |
|  |  |
| Guselkumab | 1593700 |
|  | 1593709 |
|  | 1593711 |
|  |  |
| Risankizumab | 1511348 |
|  | 1511425 |
|  | 1537579 |
|  | 1537580 |
|  |  |
| Brodalumab | 1592517 |
|  |  |
| Certolizumab pegol | 19129993 |
|  | 912263 |
|  | 912267 |
|  | 912268 |
|  |  |
| Adalimumab | 1718324 |
|  | 19127196 |
|  | 19127197 |
|  | 19133086 |
|  | 19133087 |
|  | 35604490 |
|  | 35604492 |
|  | 36249550 |
|  | 36249660 |
|  | 36879869 |
|  | 45777078 |
|  | 45777079 |
|  | 45774624 |
|  | 46275181 |
|  | 46275389 |
|  | 46275565 |
|  | 46275567 |
|  | 46275867 |
|  | 740227 |
|  | 740228 |
|  | 1119119 |
|  | 1119155 |
|  | 1592184 |
|  | 1592185 |
|  | 1592186 |
|  | 1592192 |
|  | 1592193 |
|  | 1592626 |
|  | 1592628 |
|  | 1592637 |
|  | 1592638 |
|  | 1592709 |
|  | 1592710 |
|  | 1594200 |
|  | 1594202 |
|  |  |
| Secukinumab | 46234067 |
|  | 46234069 |
|  | 45892883 |
|  | 45892886 |
|  | 45892890 |
|  | 45892892 |
|  |  |
| Infliximab | 937368 |
|  | 937369 |
|  | 1592762 |
|  | 1592767 |
|  | 19078524 |
|  | 37498070 |
|  | 46275593 |
|  | 46275595 |
|  | 41205480 |
|  | 42629515 |
|  | 42629519 |
|  |  |
| Usteskinumab | 46234167 |
|  | 46234168 |
|  | 46234169 |
|  | 46234170 |
|  | 46234171 |
|  | 44107253 |
|  | 40161532 |
|  | 40160959 |
|  | 40160960 |
|  | 40160987 |
|  | 40160988 |
|  | 1718931 |
|  | 1718933 |
|  | 1718934 |
|  |  |
| Anakinra | 1114375 |
|  | 1114379 |
|  | 1114380 |
|  |  |
| Apremilast | 44816127 |
|  | 44816128 |
|  | 44816129 |
|  | 44816130 |
|  | 44816294 |
|  | 46221784 |
|  |  |
| Upadacitinib | 1361580 |
|  | 1361707 |
|  | 1361708 |
|  |  |
| Roflumilast | 964190 |
|  | 40236899 |
|  | 40236900 |
|  |  |
| Etanercept | 19131494 |
|  | 19132639 |
|  | 46234061 |
|  | 46234063 |
|  | 37003587 |
|  | 19064171 |
|  | 19064172 |
|  | 19065564 |
|  | 19068245 |
|  | 1151789 |
|  | 1151884 |
|  | 1151885 |
|  |  |
| Clindamycin | 997881 |
|  | 997892 |
|  | 997899 |
|  | 997934 |
|  | 997937 |
|  | 997955 |
|  | 997973 |
|  | 997979 |
|  | 997980 |
|  | 997995 |
|  | 997996 |
|  | 997997 |
|  | 998000 |
|  | 998001 |
|  | 998012 |
|  | 19044180 |
|  | 19030822 |
|  | 19075425 |
|  | 19075429 |
|  | 19075430 |
|  | 19085270 |
|  | 19126573 |
|  | 19127878 |
|  | 35142000 |
|  | 40171747 |
|  | 40161186 |
|  | 40161196 |
|  | 35604117 |
|  | 35604118 |
|  | 35604327 |
|  | 35604328 |
|  | 35604329 |
|  | 35604330 |
|  |  |
| Erythromycin | 1746940 |
|  | 1747210 |
|  | 1747232 |
|  | 1836521 |
|  | 1747484 |
|  | 1747361 |
|  | 1747396 |
|  | 1747426 |
|  | 19018976 |
|  | 19043181 |
|  | 19006683 |
|  | 19080738 |
|  | 19122215 |
|  | 19125424 |
|  | 19125426 |
|  | 19125430 |
|  | 35603400 |
|  | 35603401 |
|  | 19109726 |
|  | 19113150 |
|  | 19076957 |
|  | 19076958 |
|  | 40166627 |
|  | 40166629 |
|  | 40166631 |
|  | 19031216 |
|  | 19031217 |
|  | 19031219 |
|  | 19031220 |
|  |  |
| Moxifloxacin | 19129337 |
|  | 19129961 |
|  | 1716903 |
|  | 1716904 |
|  | 1716905 |
|  | 1716908 |
|  | 36888537 |
|  | 40165695 |
|  |  |
| Tetracycline | 1836948 |
|  | 1836953 |
|  | 1836975 |
|  | 1836978 |
|  | 1837001 |
|  | 1837004 |
|  | 19019852 |
|  | 45775914 |
|  |  |
| Ertapenem | 35606486 |
|  | 35606487 |
|  | 35606488 |
|  | 35606491 |
|  | 1717963 |
|  |  |
| Ceftriaxone | 36894722 |
|  | 46287316 |
|  | 46287321 |
|  | 46287324 |
|  | 46287328 |
|  | 46287330 |
|  | 46287335 |
|  | 19074975 |
|  | 19074977 |
|  | 1777806 |
|  |  |
| Doxycyline | 1510384 |
|  | 1738521 |
|  | 1738579 |
|  | 1738611 |
|  | 1738670 |
|  | 1738722 |
|  | 1738731 |
|  | 1738756 |
|  | 19025855 |
|  | 19029685 |
|  | 19029717 |
|  | 19029718 |
|  | 19029720 |
|  | 19127264 |
|  | 19129140 |
|  | 19129143 |
|  | 19129364 |
|  | 19113415 |
|  | 19113417 |
|  | 19114563 |
|  | 19121894 |
|  | 19082954 |
|  | 19108021 |
|  | 19096551 |
|  | 19076752 |
|  | 19076753 |
|  | 19076754 |
|  | 36250178 |
|  | 19133457 |
|  | 19134051 |
|  | 35201899 |
|  | 35201900 |
|  | 40163861 |
|  | 40171393 |
|  | 40171394 |
|  | 40221290 |
|  | 40239157 |
|  | 40239159 |
|  | 46233702 |
|  | 46233710 |
|  | 46233711 |
|  | 46233757 |
|  | 46233988 |
|  | 46233989 |
|  | 46234412 |
|  | 46234415 |
|  | 46234419 |
|  | 46234523 |
|  | 46234525 |
|  | 46287568 |
|  | 45776799 |
|  | 43526701 |
|  | 19130120 |
|  | 19130162 |
|  | 19130459 |
|  |  |
| Minocycline | 793909 |
|  | 1708880 |
|  | 1708903 |
|  | 1708904 |
|  | 1708959 |
|  | 1708960 |
|  | 1708961 |
|  | 1709001 |
|  | 1709040 |
|  | 40163930 |
|  | 40227584 |
|  | 19002938 |
|  | 19002939 |
|  | 19058685 |
|  | 19035318 |
|  | 19035320 |
|  | 19035325 |
|  | 19035327 |
|  | 19102606 |
|  |  |
| Ofloxacin | 923081 |
|  | 19081762 |
|  |  |
| Rifampin | 19006753 |
|  | 19019700 |
|  | 19019811 |
|  | 19025865 |
|  | 1763204 |
|  | 1763294 |
|  |  |
| Tigecycline | 19122045 |
|  | 1742434 |
|  |  |
| Demeclocycline | 40173353 |
|  |  |
| Triamcinalone | 740264 |
|  | 789932 |
|  | 903963 |
|  | 40085179 |
|  | 40163406 |
|  | 40233205 |
|  | 40233219 |
|  | 40234030 |
|  | 40234031 |
|  | 40234037 |
|  | 40234038 |
|  | 40234046 |
|  | 40234053 |
|  | 40234056 |
|  | 40234801 |
|  | 40234818 |
|  | 40234819 |
|  | 40919692 |
|  | 40950844 |
|  | 42629018 |
|  |  |
| Metronidazole | 19021080 |
|  | 19035112 |
|  | 19035114 |
|  | 19035115 |
|  | 19035116 |
|  | 19035117 |
|  | 19044204 |
|  | 19080187 |
|  | 19081680 |
|  | 19125484 |
|  | 1707164 |
|  | 1707346 |
|  | 1707348 |
|  | 1707403 |
|  | 1707475 |
|  |  |
| Dapsone | 1711759 |
|  | 1711761 |
|  | 1711792 |
|  |  |
| Psoriasis | 9014002 |
| Rheumatoid Arthritis | 69896004 |
| Hypertension | 38341003 |
| Diabetes mellitus, type 2 | 44054006 |
| Spondyloarthritis | 784332006 |
| Polycystic ovary syndrome | 237055002 |
| Crohn's disease | 34000006 |
| Ulcerative colitis | 64766004 |
| Anxiety disorder | 197480006 |
| Depressive disorder | 35489007 |
|  |  |
| In the past 7 days, how would you rate your pain on average? | 1585747 |
|  |  |
| In the past 7 days, how would you rate your fatigue? | 1585748 |
| Average Fatigue 7 Days: None | 1585749 |
| Average Fatigue 7 Days: Mild | 1585750 |
| Average Fatigue 7 Days: Moderate | 1585751 |
| Average Fatigue 7 Days: Severe | 1585752 |
| Average Fatigue 7 Days: Very Severe | 1585753 |
|  |  |
| In general, please rate how well you carry out your usual social roles. (This includes activities at home, at work and in your community, and responsibilities as a parent, child, spouse, employee, friend, etc.) | 1585754 |
| General Social: Excellent | 1585755 |
| General Social: Very Good | 1585756 |
| General Social: Good | 1585757 |
| General Social: Fair | 1585758 |
| General Social: Poor | 1585759 |
|  |  |
| In general, how would you rate your satisfaction with your social activities and relationships? | 1585735 |
| Social Satisfaction: Excellent | 1585736 |
| Social Satisfaction: Very Good | 1585737 |
| Social Satisfaction: Good | 1585738 |
| Social Satisfaction: Fair | 1585739 |
| Social Satisfaction: Poor | 1585740 |
|  |  |
| To what extent are you able to carry out your everyday physical activities such as walking, climbing stairs, carrying groceries, or moving a chair? | 1585741 |
| Everyday Activities: Completely | 1585742 |
| Everyday Activities: Mostly | 1585743 |
| Everyday Activities: Moderately | 1585744 |
| Everyday Activities: A Little | 1585745 |
| Everyday Activities: Not At All | 1585746 |
|  |  |
| In general, would you say your quality of life is: | 1585717 |
| General Quality: Excellent | 1585718 |
| General Quality: Very Good | 1585719 |
| General Quality: Good | 1585720 |
| General Quality: Fair | 1585721 |
| General Quality: Poor | 1585722 |
