# Supplementary material for: Understanding the evolving treatment landscape of hidradenitis suppurativa: An analysis of All of Us
Source: PLoS One. 2025 Aug 22;20(8):e0331032. doi: 10.1371/journal.pone.0331032 (PMC12373187; doi:10.1371/journal.pone.0331032)
Supplement: S2 Table — (DOCX) [file pone.0331032.s002.docx]

**S2 Table – Summary data for HS population within the AoU program**

|  |  | Proportion of Patients (%) |
| --- | --- | --- |
| Total (n = 2636) | |  |
| Male (%) | | 18.55 |
| Female (%) | | 80.54 |
| Race/Ethnicity | |  |
|  | *White/Caucasian* | 39.11 |
|  | *Black/African American* | 31.90 |
|  | *Hispanic* | 16.62 |
|  | *Other/Unknown* | 12.37 |
| Age |  |  |
|  | *<18 yrs* | 22.66 |
|  | *18 to 64 yrs* | 73.47 |
|  | *>65 yrs* | 3.87 |
| Drug Class* | |  |
|  | *Antibiotics* | 72.00 |
|  | *Immunosuppressives* | 48.48 |
|  | *Biologics* | 10.43 |
|  | *Small Molecule Inhibitors* | 2.20 |
|  |  |  |
| * patients may have received more than one drug class | | |
