# Supplementary figures and images for: Understanding the evolving treatment landscape of hidradenitis suppurativa: An analysis of All of Us
Source: PLoS One. 2025 Aug 22;20(8):e0331032. doi: 10.1371/journal.pone.0331032 (PMC12373187; doi:10.1371/journal.pone.0331032)

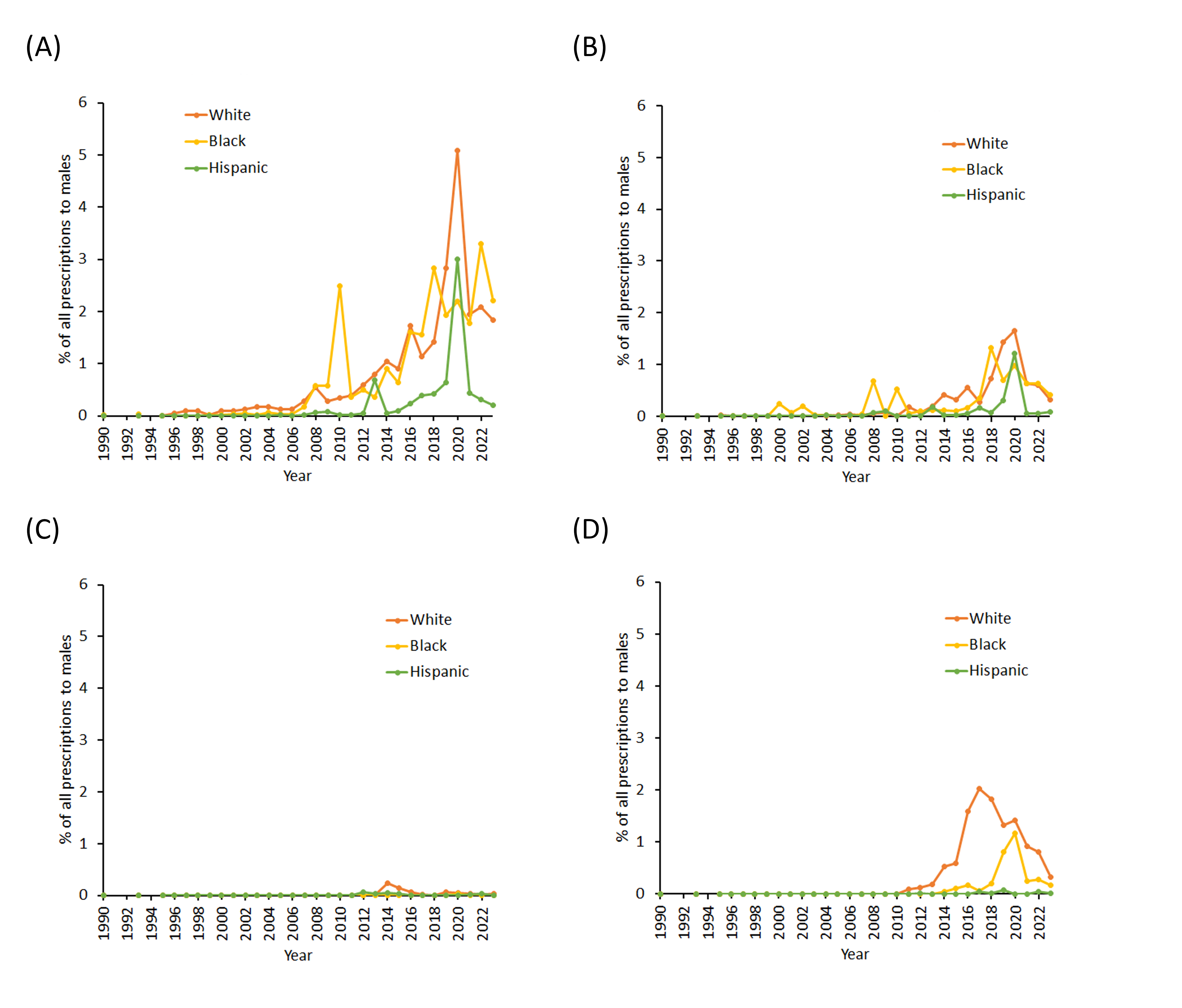

Supplement: S1 Fig — (A) Antibiotics, (B) Immunosuppressives, (C) Small molecule inhibitors and (D) Biologics. (TIF) [file pone.0331032.s003.tif]
